# Electrodeposition–Assisted Assembled Multilayer Films of Gold Nanoparticles and Glucose Oxidase onto Polypyrrole-Reduced Graphene Oxide Matrix and Their Electrocatalytic Activity toward Glucose

**DOI:** 10.3390/nano8120993

**Published:** 2018-12-01

**Authors:** Baoyan Wu, Shihua Hou, Yongyong Xue, Zhan Chen

**Affiliations:** 1 MOE Key Laboratory of Laser Life Science & Institute of Laser Life Science, College of Biophotonics, South China Normal University, Guangzhou 510631, China; wubaoyan@scnu.edu.cn (B.W.); xueyongyongscnu@163.com (Y.X.); 2 School of Electronic and Information Engineering, South China University of Technology, Guangzhou 510640, China; 3 College of Life Sciences, South China Normal University, Guangzhou 510631, China; chenzhanscnu@163.com

**Keywords:** polypyrrole, graphene, gold nanoparticles, electrodeposition, self-assembly, biosensor

## Abstract

The study reports a facile and eco-friendly approach for nanomaterial synthesis and enzyme immobilization. A corresponding glucose biosensor was fabricated by immobilizing the gold nanoparticles (AuNPs) and glucose oxidase (GOD) multilayer films onto the polypyrrole (PPy)/reduced graphene oxide (RGO) modified glassy carbon electrode (GCE) via the electrodeposition and self-assembly. PPy and graphene oxide were first coated on the surface of a bare GCE by the electrodeposition. Then, AuNPs and GOD were alternately immobilized onto PPy-RGO/GCE electrode using the electrodeposition of AuNPs and self-assembly of GOD to obtain AuNPs-GOD multilayer films. The resulting PPy-RGO-(AuNPs-GOD)_n_/GCE biosensors were used to characterize and assess their electrocatalytic activity toward glucose using cyclic voltammetry and amperometry. The response current increased with the increased number of AuNPs-GOD layers, and the biosensor based on four layers of AuNPs-GOD showed the best performance. The PPy-RGO-(AuNPs-GOD)_4_/GCE electrode can detect glucose in a linear range from 0.2 mM to 8 mM with a good sensitivity of 0.89 μA/mM, and a detection limit of 5.6 μM (S/N = 3). This study presents a promising eco-friendly biosensor platform with advantages of electrodeposition and self-assembly, and would be helpful for the future design of more complex electrochemical detection systems.

## 1. Introduction

Diabetes is a growing public health problem and its complications, rather than the disease itself, are harmful for human body [1]. In medicine, diabetes is diagnosed mainly by detecting the concentration of glucose in the blood/urine [2]. Hence, glucose detection and quantification has received much attention. Until now, numerous techniques, such as chromatography [3,4], colorimetric [5,6], and electrochemistry [7,8] have been proposed for the determination of glucose. Although each glucose detection system has strengths and weaknesses, electrochemical methods have gained wide acceptance for analyses and are preferred over other techniques because of their simplicity, relatively low-costs, easy manufacturing and industrialization, and portable instruments [7,8,9,10]. Of the various glucose electrochemical detection methods, glucose oxidase modified electrodes play a crucial role in the detection of glucose [7,9,11]. Glucose oxidase is one of the most extensively researched enzymes for the construction of glucose enzymatic biosensors [12].

Enzyme immobilization matrix is a key component in biosensor fabrication. It can deeply influence the electrocatalytic activity of enzymatic sensors because of its improved recovery ability from the reaction systems for catalyst reuse as compared to homogeneously solubilized enzyme molecules [13,14,15]. With the continuous development of nanotechnology, close attention was taken in preparing the hybrid nanostructures as enzyme immobilization matrix [16,17,18]. For example, Wang et al., fabricated a transistor/glucose biosensor based on reduced graphene oxide (RGO) and polypyrrole (PPy) hybrid nanowires active layer [19]. Xue et al. used a ternary PPy/RGO/gold nanoparticles (AuNPs) nanocomposite to immobilize glucose oxidase [20]. The as-prepared biosensors exhibited good electrocatalytic property towards O_2_ or H_2_O_2_. PPy/RGO/AuNPs nanocomposites had already been applied in enzymatic biosensors and obtained satisfactory results.

PPy/RGO/AuNPs nanocomposites have typically been prepared by chemical or chemical–electrochemical routes. Although these methods have advantages of large-scale and high yield, they frequently use excessive reducing agents resulting in contaminated nanocomposites, as well as enzyme denaturation and inactivation [21,22,23]. Recently, we reported a facile PPy/RGO/AuNPs fabrication on a glassy carbon electrode by drop-casting and electrodeposition consisting of the electrooxidation of pyrrole monomer and simultaneous electroreduction of graphene oxide and auric ions (Au^3+^) in aqueous solution [24]. Despite the facile eco-friendly synthesis method, this work still suffers from film thickness side effects, because the drop-casting method lack control over film thickness [25].

Enzyme immobilization method is another critical process in enzymatic biosensor development. The immobilization of enzymes onto the electrode not only influences enzyme activity, but can also maintain the structural integrity of the enzyme for longer, which plays an important role in the overall performance of enzymatic biosensors [26,27]. Layer-by-layer (LBL) self-assembly has been extensively studied and proved. LBL assembly is a versatile approach to construct various biosensors with some obvious advantages owing to its operational controllability and simplicity [28,29,30,31]. It is important to note that the representation of the LBL assembly as a multilayer build-up based on a variety of interaction, including electrostatic attraction, hydrophobic attraction, covalent bonding, hydrogen bonding, and host-guest et al. [32,33]. More importantly, LBL assembly can enhance the sensitivity of the biosensor. Xue et al., built a graphene–gold nanorod hybrid architecture via LBL for electrochemical biosensors, which exhibited the highest sensitivity compared to the graphene oxide, cysteine-graphene oxide and graphene–gold nanorod coated electrodes [34]. Voelcker et al., reported that an electrochemical immune-sensor, prepared by self-assembly of peptides, afforded higher sensitivities for cardiac troponin I than those prepared by the chemisorption of alkane thiolated compounds [35].

Influenced by previous studies, this study focused on proposing a facile and environmentally friendly approach for PPy/RGO/AuNPs synthesis and glucose oxidase immobilization. The as-obtained amperometric glucose biosensor, based on the novel enzyme immobilization matrix, consisted of the electrodeposition–assisted assembled multilayer films of AuNPs-GOD and the electrodeposited PPy-RGO film. The amperometric glucose biosensor was developed and characterized by cyclic voltammetry and amperometry.

## 2. Materials and Methods

### 2.1. Chemicals

Glucose Oxidase from *Aspergillus niger* (GOD, EC 1.1.3.4, 100–250 U/mg) was purchased from Sigma-Aldrich Co. (Saint Louis, MO, USA). Graphene oxide (GO, 1 mg/mL, solvent: water) was purchased from Xianfeng NANO material Tech Co. Ltd. (Nanjing, China). HAuCl_4_·4H_2_O and pyrrole monomer were purchased from J&K Scientific Ltd. (Beijing, China). Glucose, ascorbic acid, 4-acetamidophenol and uric acid were purchased from Aladdin Industrial Inc (Shanghai, China). Phosphate-buffered saline (0.1 M PBS, pH 7.0) buffer was employed as a supporting electrolyte. All electrochemical experiments were performed in PBS at room temperature, approximately 25 °C.

### 2.2. Apparatus and Measurements

Electrochemical measurements were carried out with CHI800C electrochemical analyzer (Chen Hua Instruments, Shanghai, China) with a conventional three-electrode system, in which a glassy carbon electrode (GCE), a saturated calomel electrode (SCE) and a platinum wire electrode served as the working, reference, and auxiliary electrodes, respectively. The surface morphological features of PPy-GO and PPy-RGO-AuNPs were characterized using ZEISS Ultra 55 scanning electron microscopy (Carl Zeiss, Oberkochen, Germany).

### 2.3. Configuration of the Amperometric Glucose Biosensor

PPy-RGO-(AuNPs-GOD)_n_/GCE was prepared according to the following steps:

Step 1: The bare GCE electrode was polished with chamois leather containing 0.05 μm alumina slurry, rinsed thoroughly with water, sonicated in ethanol, and dried at room temperature.

Step 2: GO (0.5 mg/mL) were sonicated in pyrrole monomer solution (0.3 M), centrifuged at 10,000 rpm for 10 min, and collected the supernatant. Then, the cleaned bare GCE was immersed into the supernatant, and a continuous cyclic voltammetric sweep of 10 cycles with potential ranging from 0 V to 0.8 V versus SCE was performed at a scan rate of 25 mV/s for the electrooxidation of pyrrole monomer and simultaneous immobilization of polypyrrole (PPy) and GO onto the electrode surface. The as-obtained electrode was regard as PPy-GO/GCE.

Step 3: PPy-GO/GCE was immersed into PBS containing 6.5 mM HAuCl_4_·4H_2_O. Then, a continuous cyclic voltammetric sweep of 6 cycles with potential ranging from 0 V to −1.5 V versus SCE was performed at a scan rate of 25 mV/s to ensure the simultaneous electroreduction of both GO and HAuCl_4_ on the PPy-GO/GCE surface, which was regarded as PPy-RGO-AuNPs/GCE electrode.

Step 4: PPy-RGO-AuNPs/GCE was immersed in GOD (2 mg/mL) for 6 hours at 4 °C to immobilize GOD, and then thoroughly rinsed with PBS for dissociating the weak adsorption, which was regarded as the one layer of AuNPs-GOD multilayer films modified PPy-RGO/GCE electrode, PPy-RGO-(AuNPs-GOD)_1_/GCE.

Step 5: The different layers of multilayer films (AuNPs-GOD)_n_ modified PPy-RGO/GCE electrodes, PPy-RGO-(AuNPs-GOD)_n_/GCE were obtained by repeating (n − 1) times of the electro-deposition of AuNPs and self-assembly of GOD.

## 3. Results and Discussion

### 3.1. Glucose Biosensor Based on Polypyrrole, Reduced Graphene Oxide, Nanogold and Glucose Oxidase

**Figure 1:**
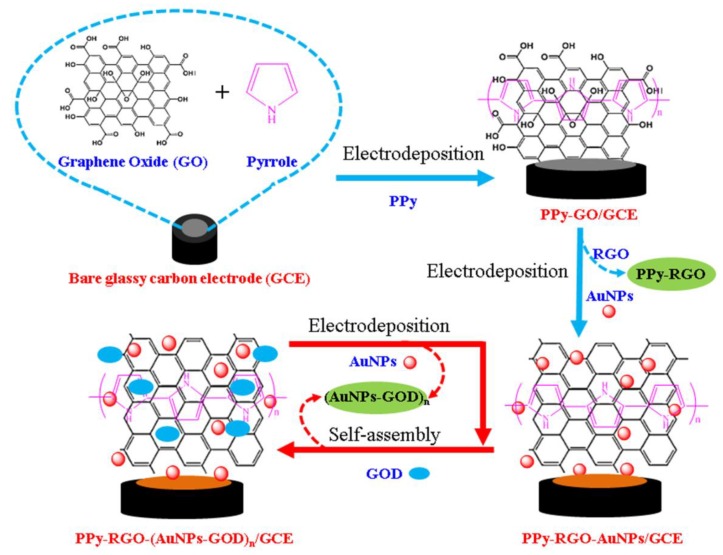
Schematic illustration of a glucose biosensor using the polypyrrole and reduced graphene oxide (PPy-RGO), and gold nanoparticles and glucose oxidase (AuNPs-GOD) multilayer films as the sensitive layer fabricated by the electrodeposition and self-assembly.

The fabrication process of the glucose biosensor based on the electrodeposition–assisted assembled multilayer films of AuNPs and glucose oxidase (AuNPs-GOD)_n_, and the polypyrrole/reduced graphene oxide film (PPy-RGO) modified GCE is shown in Figure 1. PPy and graphene oxide (GO) was first assembled on the bare GCE surface via the electrooxidation of pyrrole to obtain PPy-GO/GCE electrode. Second, AuNPs were electro-reductively deposited on the PPy-GO/GCE electrode surface with the electrochemical reduction of GO immobilized on the PPy-GO/GCE to obtain PPy-RGO-AuNPs/GCE. Third, GOD was self-assembled on PPy-RGO-AuNPs/GCE surface mainly via the interaction between AuNPs and GOD to obtain the PPy-RGO-(AuNPs-GOD)_1_/GCE electrode. It has been reported that AuNPs can bind strongly to the biomolecule surface through covalent bonds with the functional groups such as –NH_2_, SH, and –CN, and it can provide a suitable microenvironment for biomolecules, such as enzyme and nucleic acids [11,36]. Finally, different layers of AuNPs-GOD multilayer films were built up on the PPy-RGO/GCE surface by repeating the process of electrodeposition of AuNPs and self-assembly of GOD to obtain PPy-RGO-(AuNPs-GOD)_n_/GCE electrode. Herein, this proposed amperometric glucose biosensor, using the PPy-RGO and AuNPs-GOD multilayer films as the sensitive layer, was developed by the eco-friendly electrodeposition and self-assembly without redox reagent, separation procedure, crosslinking or adhesive agents. The possible working mechanisms may involve the following biochemical reactions:

GOD(FAD) + β-d-glucose → GOD(FADH_2_) + d-glucono-1,5-lactone (1)

GOD(FADH_2_) → GOD(FAD) + 2H^+^ + 2e^−^ (2)

GOD(FADH_2_) + O_2_ → GOD(FAD) + H_2_O_2_ (3)

H_2_O_2_ → O_2_ + 2 H^+^ + 2e^−^ (4)

When GOD (FAD) catalyzes glucose oxidation, GOD catalyzes glucose to form gluconolactone, and GOD itself is reduced to GOD (FADH_2_). Then, GOD (FADH_2_) is oxidized to GOD (FAD), which can be achieved in two ways: by oxygen re-oxidation back to GOD (FAD) and producing hydrogen peroxide (H_2_O_2_) in the process (first generation biosensors), or via direct electron transfer (DET) between FADH_2_ and electrodes (third generation biosensors) [37,38]. Thus, either the electroactivity of H_2_O_2_ or the direct response current of FADH_2_ can be used as a measure of glucose in this study.

**Figure 2:**
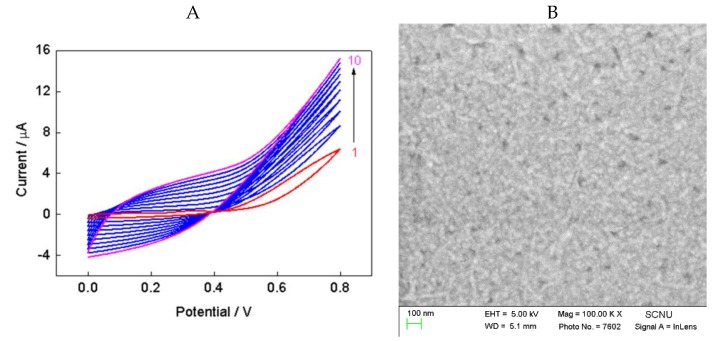
Cyclic voltammograms (CVs) of bare glassy carbon electrode (GCE) in pyrrole/graphene oxide solution for 10 cycles with a scan rate of 25 mV/s (**A**) and typical SEM image of polypyrrole (PPy)/graphene oxide (GO) (**B**).

**Figure 3:**
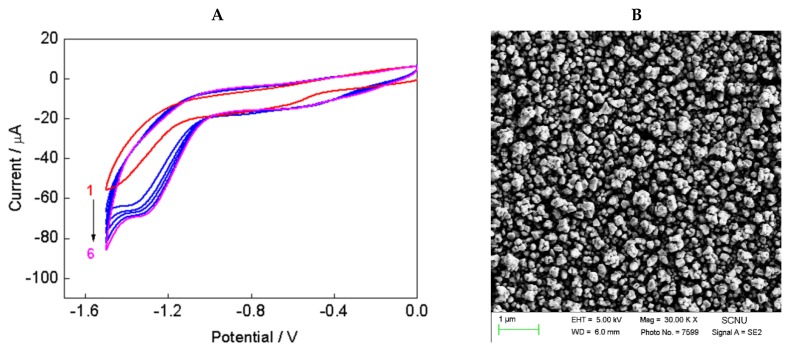
CVs of PPy-GO/GCE electrode in HAuCl_4_ aqueous solution for 6 cycles with a scan rate of 25 mV/s (**A**), and the typical SEM image of PPy-RGO-AuNPs (**B**).

The electrochemical procedure for the preparation of polypyrrole, reduced graphene oxide, and AuNPs was prepared using literature research [24], with some changes. Here the electrochemical oxidation and the electrochemically reduction are separately performed to avoid drop casting side effects. The electrochemically oxidation of pyrrole with a potential range of 0 V–0.8 V versus SCE and the morphology of PPy-GO film are shown in Figure 2. Cyclic voltammograms (Figure 2A) illustrated the continuous electrochemical oxidation of pyrrole, and PPy-GO presented a typical wrinkled and crumpled shape (Figure 2B). As reported in the literature [39,40], pyrrole can be oxidized at 0.8 V. Higher than 0.8 V may potentially lead to the formation of overoxidized polypyrrole films with poor adherence at the electrode surface. The electrochemical reduction of HAuCl_4_ and GO with a potential range of −1.5 V–0 V versus SCE, and the morphology of PPy-RGO-AuNPs, are shown in Figure 3. Cyclic voltammograms (Figure 3A) illustrated the continuous electrochemical reduction of HAuCl_4_ and the surface oxygen groups of GO as well as liberating AuNPs and reduced graphene oxide, which was in agreement with previous reports [21,41]. Clearly, the surface morphological feature of PPy-RGO-AuNPs film (Figure 3B) was remarkably different from the PPy-GO film. Many AuNPs were electrochemically deposited in uniform manner, and only a few AuNPs formed larger clusters. The color of PPy-RGO-AuNPs/GCE was visible through a color change of PPy-GO/GCE from clear black to dark brown. The electrochemical deposition process is a potential method for nanomaterials modified electrodes of various active surfaces without redox reagent and separation procedures [42,43]. Thus, all electrochemically conducting surfaces can be covered by PPy, RGO and AuNPs in uniform manner, which can improve the performance of biosensors.

### 3.2. The Electrochemical Properties of PPy-RGO-AuNPs Nanocomposite

**Figure 4:**
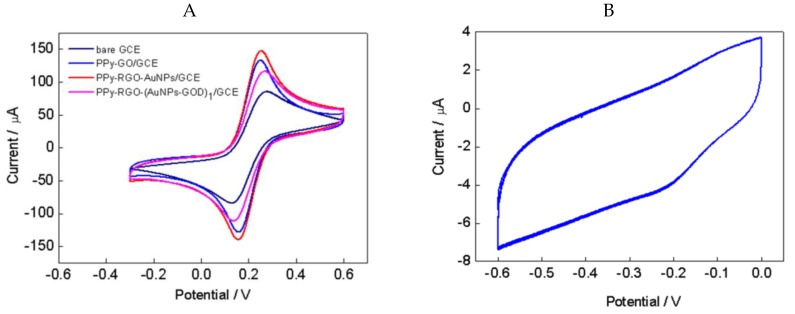
(**A**) CVs of the electrode at different stages: bare GCE; PPy-GO/GCE; PPy-RGO-AuNPs/GCE; and PPy-RGO-(AuNPs-GOD)_1_/GCE. Supporting electrolyte, 0.1 M KCl containing 5mM [Fe (CN)_6_]^3−/4−^ (1:1). (**B**) CVs of PPy-RGO-(AuNPs-GOD)_1_/GCE recorded in pH 7.0 phosphate buffer solutions. Scan rate, 50 mV/s.

The electron transfer properties of various modified electrodes (bare GCE, PPy-GO/GCE, PPy-RGO-AuNPs/GCE, PPy-RGO-(AuNPs-GOD)_1_/GCE) were studied using cyclic voltammetry in the KCl solution containing [Fe(CN)_6_]^3−/4−^ redox couple with a san rate of 50 mV/s. From the results shown in Figure 4A, the oxidation and reduction peaks were observed at bare GCE, which are related to the electrochemical conversions of Fe (II) to Fe (III) and vice versa. When the GCE electrode was modified with PPy-GO and AuNPs, the peak current of redox had a significant increase, while the peak separation had a decrease compared with bare GCE, confirming the contribution of PPy-GO, RGO and AuNPs. After GOD was adsorbed on PPy-RGO-AuNPs/GCE surface, the resulting PPy-RGO-(AuNPs-GOD)_1_/GCE electrode decreased peak current and increased peak separation, mainly due to the electrical insulating properties of GOD [44].

Direct electron transfer (DET) of GOD was investigated at a PPy-RGO-(AuNPs-GOD)_1_/GCE electrode in PBS using cyclic voltammetry with a san rate of 50 mV/s. As can be seen from Figure 4B, no GOD redox peak corresponding to the conversion between GOD (FADH_2_) and GOD (FAD) could be observed, indicating that reaction (2)/DET of GOD did not taking place in the present PPy-RGO-AuNPs-GOD-based glucose biosensor. This may be attributed to the fact that the active redox center (FAD/FADH_2_) of GOD is deeply embedded in a protective protein shell, resulting in the direct electron transfer between GOD and electrode (DET-GOD) being difficult to realize [45]. Although DET is important for the development of electrochemical biosensor, previous studies have reported DET-GOD will lose their native bioactivity owing to the great change in the GOD structure [46,47]. Wooten et al., reported GOD adsorbed on CNT yielded a pair of GOD redox peak, but the anodic peak did not increase in the presence of glucose in an O_2_-free solution [48]. It has also been demonstrated that DET-GOD is without value in certain mediator-free applications [49]. So the electroactivity of glucose oxidase product H_2_O_2_ (Reactions (3) and (4)) was still chosen as the measure of glucose for PPy-RGO-(AuNPs-GOD)_n_/GCE in the study. Meanwhile, the result further revealed that our proposed method could maintain the structural integrity of glucose oxidase.

### 3.3. Electrochemical Performance of PPy-RGO-(AuNPs-GOD)_n_/GCE Electrode

**Figure 5:**
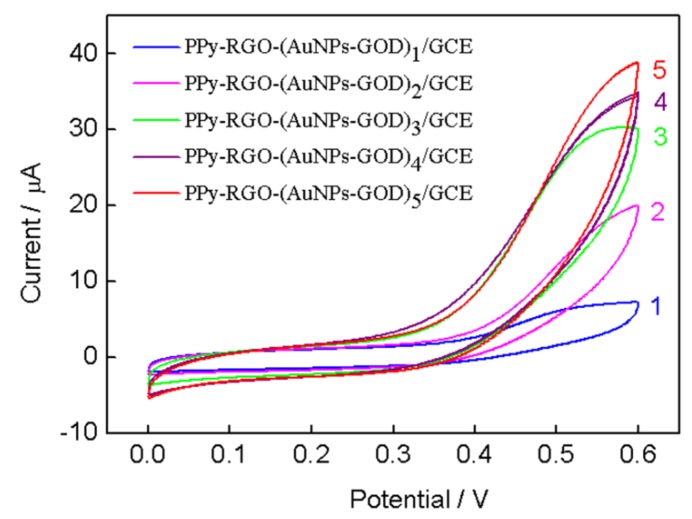
CVs of different layers of AuNPs-GOD multilayer films modified PPy-RGO/GCE in 10 mM glucose. The layer number of multilayer films was indicated on the graph. Scan rate, 50 mV/S.

The immobilization amount of GOD on the electrode has a significant effect on the biosensor response. PPy-RGO-(AuNPs-GOD)_n_/GCE electrodes based on the different layers of AuNPs-GOD multilayer films were fabricated by the combination of the electrodeposited AuNPs and self-assembled GOD, and were characterized by cyclic voltammogram in 10mM glucose. As shown in Figure 5, curves 1–5 displayed cyclic voltammograms of 1–5 layers of AuNPs-GOD multilayer films modified PPy-RGO/GCE electrode, respectively. The response current of the resulting glucose biosensors increased with the increase of the layer number of AuNPs-GOD films. From one to three layers, the current approximately uniformly increased with an increase of more than 10 μA, and then the current slowly increased after three layers. So the PPy-RGO-(AuNPs-GOD)_4_/GCE electrode was chosen for further amperometric investigation.

**Figure 6:**
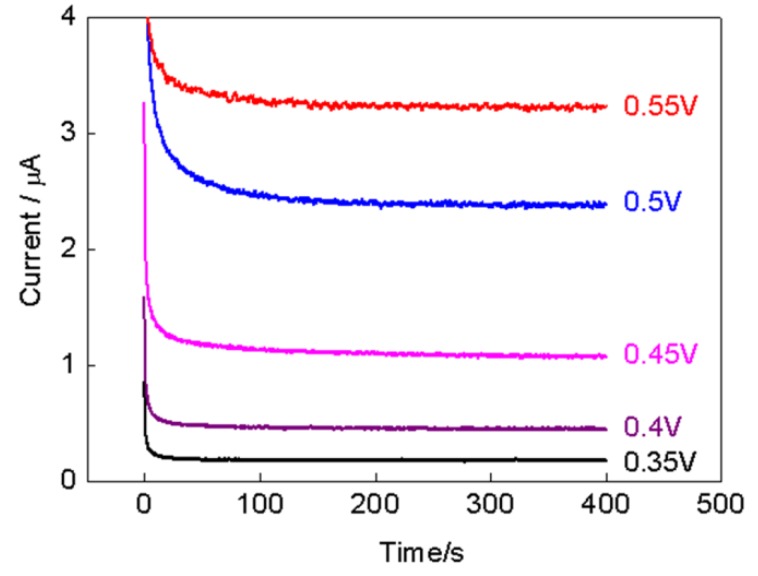
Current-time curves for PPy-RGO-(AuNPs-GOD)_4_/GCE in 4 mM glucose at different applied potentials from 0.35 V to 0.55 V versus SCE.

The applied potential has an important influence over the electrochemical biosensor performance. The effect of applied potential on the response current of PPy-RGO-(AuNPs-GOD)_4_/GCE electrode was explored by varying the potential from 0.35 V to 0.55 V versus SCE in the 4 mM glucose. The obtain amperometric results are shown in Figure 6. The applied potential obviously influenced the response current, and the current increased with the increase of applied potential. By considering the sensitivity and selectivity, an applied potential of 0.5 V versus SCE was chosen as the working potential.

**Figure 7:**
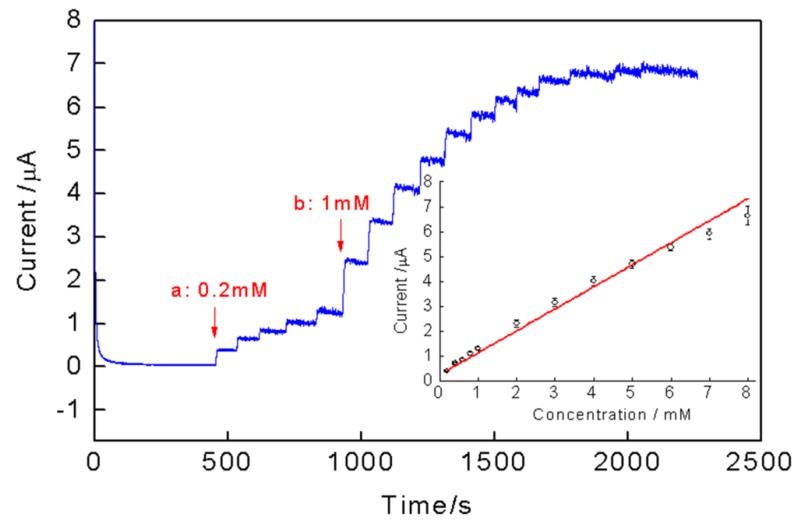
Typical current-time curve recorded at a PPy-RGO-(AuNPs-GOD)_4_/GCE during the addition of different concentration glucose (0.2 mM–13 mM) at a potential of 0.5 V versus SCE. The arrows “a” and “b” indicated each increased glucose concentration is 0.2 mM and 1 mM, respectively. Inset: the calibration curve of the electrode with glucose concentration ranged from 0.2 mM to 8 mM. Error bar = standard deviation (n = 5).

Figure 7 shows the typical current-time curve of PPy-RGO-(AuNPs-GOD)_4_/GCE to the successive additions of different concentrations of glucose to PBS at a potential of 0.5 V versus SCE under gentle magnetic stirring. After recording a baseline current in a stirred solution, 0.2 mM glucose was first added five times, followed by the addition of 1 mM glucose 12 times. The glucose concentration is from 0.2 mM to 13 mM. As the glucose was added into the stirring buffer solution, the biosensor responded rapidly to the substrates, which achieved 95% of the steady-state current within 5 seconds. With the increase of glucose concentration, the response current increased. From the corresponding calibration curve (Figure 7 inset) to see, the current and glucose concentration had a linear relationship where glucose concentration ranged from 0.2 mM to 8 mM (*R*^2^ = 0.99). The linear equation was I (μA) = 0.24 + 0.89C (mM) with an acceptable relative standard deviation of less than 7%. The sensitivity calculated from the linear calibration was 0.89 μA/mM, and a low limit of detection was estimated to be 5.6 μM (S/N = 3).

**Figure 8:**
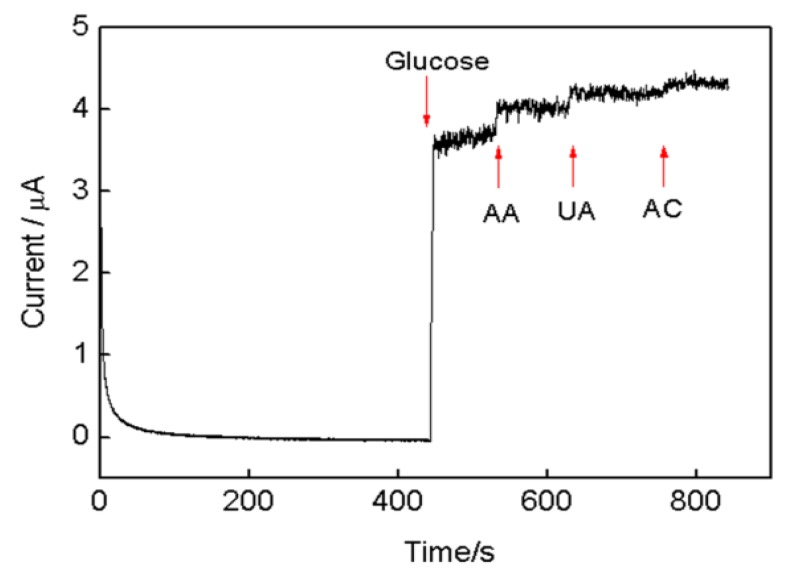
Typical current-time curve recorded at a PPy-RGO-(AuNPs-GOD)_4_/GCE during the addition of glucose, ascorbic acid (AA), uric acid (UA), and acetaminophen (AC) at a potential of 0.5 V versus SCE.

The anti-interference ability and stability were also examined. The effects of the presence of three possible interfering substances ascorbic acid (AA), uric acid (UA), and acetaminophen (AC) were examined to evaluate the selectivity of the proposed biosensor. The interfere current obtained for each interfering substance presented at a concentration of 0.1 mM was compared to that of 4 mM glucose. As shown in Figure 8, a large current reached after glucose addition, and no significant response signals were observed upon successive addition of AA, UA, and AC into the system. The small interfering current may be attributed to two aspects: First, negatively charged AuNPs and glucose oxidase can repulse the negatively charged substance. Second, hydrophobic polypyrrole and reduced graphene oxide can repulse the hydrophobic substance. Hence, there may be a repulsive effect between the PPy-RGO-(AuNPs-GOD)_4_ mulitlayer films and interfering substances, and it hinders the diffusion of interfering substances toward the electrode surface. On the contrary, H_2_O_2_ becomes an uncharged hydrophilic small molecule when compared to these interfering substances, and it can easy diffuse to the electrode surface. To assess stability, PPy-RGO-(AuNPs-GOD)_4_/GCE was stored at 4 °C after use and measured the current response for 4 mM glucose every two days. The electrode retained 90% of its original current response over two weeks, indicating a good shelf lifetime. This may be attributed to the good stability and biocompatibility of PPy, RGO and AuNPs nanocomposites, and the developed method for PPy, RGO and AuNPs synthesis and GOD immobilization with advantages of the electrodeposition and self-assembly.

## 4. Conclusions

We have developed a convenient and eco-friendly approach for the facile fabrication of the PPy/RGO/AuNPs and GOD immobilization using the electrodeposition and self-assembly. A corresponding glucose enzymatic biosensor based on the PPy-RGO film and AuNPs-GOD multilayer films was developed without redox reagent, separation procedure, crosslinking and adhesive agents. The proposed method is green and will not result in contamination of the enzyme immobilization matrix/active layer. The as-prepared PPy-RGO-(AuNPs-GOD)_4_/GCE exhibited a satisfied-performance, which would primarily be attributed to the use of PPy, RGO and AuNPs as GOD immobilization matrix, enzyme catalytic specificity and efficiency, and greatly benefit from the combination of the electrodeposition and self-assembly method. Although the biosensor may still suffer from some drawbacks, such as enzymes stability, the proposed method is sufficiently adaptable and scalable due to the above-noted advantages. The resulting biosensor can serve as a model enzymatic biosensing platform for the development of other electrochemical biosensors, which makes a promising application potential in more complex electrochemical detection systems at various electrode surfaces under environmentally friendly conditions.
